# Does (co-)variation between boldness and growth differ between sexes? A nuanced and powerful evaluation of the pace of life hypothesis

**DOI:** 10.1093/beheco/arag116

**Published:** 2026-10-21

**Authors:** Aidan M Joynson, Christa Beckmann, Peter A Biro

**Affiliations:** School of Life and Environmental Science, Deakin University, 75 Pigdons Road, Geelong, VIC 3216, Australia; School of Life and Environmental Science, Deakin University, 75 Pigdons Road, Geelong, VIC 3216, Australia; School of Science, RMIT University, 124 La Trobe Street, Melbourne, VIC 3001, Australia; School of Life and Environmental Science, Deakin University, 75 Pigdons Road, Geelong, VIC 3216, Australia

**Keywords:** animal personality, sexual selection, pace of life syndrome, hierarchical modeling, allometry, sexual dimorphism, energy allocation

## Abstract

A major goal of behavioral ecology is identifying sources of among-individual variation in behavior. Theory suggests that life history traits and personality should be correlated at the individual level, but despite much attention, it has little support. A potentially crucial aspect influencing life history and personality associations that has been overlooked is sex. Males and females of many species differ in energy requirements, energy allocation, and behavior, suggesting that sex may affect correlations between life history and personality differently; if so, not accounting for sex differences may obscure relationships. We address this possibility by intensive longitudinal sampling of boldness and growth in 66 male and 92 female pillbugs (*Armadillidium vulgare*) (total *N* = 158) using novel statistical methods to estimate sex-specific (co-)variances in a single parsimonious model. We found that females and males had similar mean boldness, similar among-individual variation in boldness and in growth rate, but females grew faster than males. We found support for a positive correlation between boldness and growth in males but no correlation in females. Differences in personality–life-history correlations between females and males may be linked to sex-specific reproductive strategies. Given the ubiquity of sex differences in life history and behavior, this sex-specific approach to studying trait integration is likely to provide novel insights in many systems that may have gone unnoticed.

## Teaser

Using the common pillbug, we show a positive connection between boldness and growth rates in male, but not in females. This suggests that personality in animals is not only related to differences in their physiology but also that such relationships can differ between males and females. We relate this finding to differences in the mating behaviour, and mate-search strategy employed by males and female pillbugs.

## Introduction

Behavioral studies have advanced in the last 2 decades through the recognition that, within populations, individuals show consistent individual differences in their behavior (often termed “personality”)—falsifying previous assumptions of total behavioral flexibility within individuals (Wilson et al. 1994; Bell et al. 2009). Several hypotheses have been proposed to explain this phenomenon, such as trade-offs between current and future reproduction (Wolf et al. 2007) and frequency-dependent behavioral strategies (Dall et al. 2004). Most of these hypotheses suggest underlying state variables as being both a driver and a consequence of individual behavioral differences.

The extended “pace of life syndrome” hypothesis (POLS) is the most prominent of the state-dependent hypotheses proposed to explain consistent individual differences in behavior. Briefly, the POLS hypothesis suggests that individuals differ in life history “pace,” whereby individuals that are highly productive (eg higher growth rate, greater offspring production) are also expected to be more bold, aggressive, and active if those behaviors contribute to resource acquisition (Stamps 2007; Biro and Stamps 2008; Réale et al. 2010). In other words, proximate physiological mechanisms (ie state dependence) underlying life history variation may constrain behavioral expression. In addition, the POLS hypothesis predicts that individuals with higher productive capacity should also have higher energy expenditure and be supported by higher resting metabolic rates (Biro and Stamps 2008, 2010; Careau et al. 2008; Réale et al. 2010; Careau and Garland 2012; Biro 2024). POLS assumes that individual variation in life history and behavior is maintained by frequency dependence (Dall et al. 2004; Wolf et al. 2008), fluctuating selection pressures (Dingemanse and Réale 2005), and/or flat fitness functions (Stamps 2007). Despite its intuitive appeal and a great many studies that have tested these ideas over the past 15 years, empirical support remains weak.

Literature reviews and meta-analyses have found little to no support for the POLS hypothesis or indeed any correlation between life history productivity, metabolism, and behavior (Niemelä and Dingemanse 2018; Royauté et al. 2018). Reasons for this lack of support might include methodological deficiencies (eg small sample sizes) and context-dependency of trait associations that are not captured in the study design (Burton et al. 2011; Niemelä and Dingemanse 2018; Pettersen et al. 2018; Mathot et al. 2019). Context-dependency refers to the possibility that correlations may vary with situations and ecological variables, requiring a more nuanced approach to making predictions and study design. Indeed, this was raised in some of the early papers outlining the POLS hypothesis (Biro and Stamps 2008, 2010; Careau and Garland 2012), but few studies have adopted a nuanced approach to address the potential for context-dependency.

A potentially crucial context-dependency that was not explicitly considered in early theory and subsequent publications testing POLS is the potential for sex-specific (co-)variation of productivity and behavior. This nuance of the POLS hypothesis suggests that different selection pressures between males and females may alter their relationship between behavior and life history (Hämäläinen et al. 2018; Immonen et al. 2018). Anisogamy and the large differences in parental investment in offspring care are selective pressures likely to generate differences in means, variances, and covariances between sexes (Bateman 1948; Schuett et al. 2010; Lehtonen et al. 2016; Somjee et al. 2022).

Across various species, males have shown greater mean activity, boldness, and exploration compared with females (*Cherax destructor*: Biro et al. 2014; *Gambusia holbrooki*: Brand et al. 2023a; *Drosophila melanogaster*: Brand et al. 2023b; *Poecilia reticulata*: Harris et al. 2010; *Deinacrida rugosa*: Kelly 2024; *Gasterosteus aculeatus*: King et al. 2013; *Ficedula hypoleuca*: Krams et al. 2014). Further, males that do not provide parental care and compete for mates via “scramble” competition could benefit from high boldness, as greater risk-taking may increase the likelihood of locating receptive females for mating opportunities. This combination would lead to a positive correlation between growth and boldness at the among-individual level in males, consistent with the “performance” model of energy management (Biro and Stamps 2008, 2010; Careau and Garland 2012). By contrast, in many species, larger females are generally more fecund, and so relatively faster-growing females may exhibit lower boldness to safeguard developing reproductive potential. This may produce a negative correlation between growth rate and boldness at the among-individual level in females, consistent with both the “asset protection” principle (Clark 1994) and the “allocation” model of energetic management (Careau and Garland 2012).

Given these examples and known differences between sexes in energy allocation, we might expect that females will be shyer in general, allocate resources toward reproduction, and show a negative correlation between behavior and productivity in contrast to that of males. In this hypothetical scenario, then the generally weak associations found between behavior and growth rate (Fig. 1a) might be explained by sex differences in correlations (Fig. 1b). Such sex differences in covariance, if present, require that data analyses fit variance components separately for each sex within a single model that also fits differences in means as a fixed factor.

**Figure 1:**
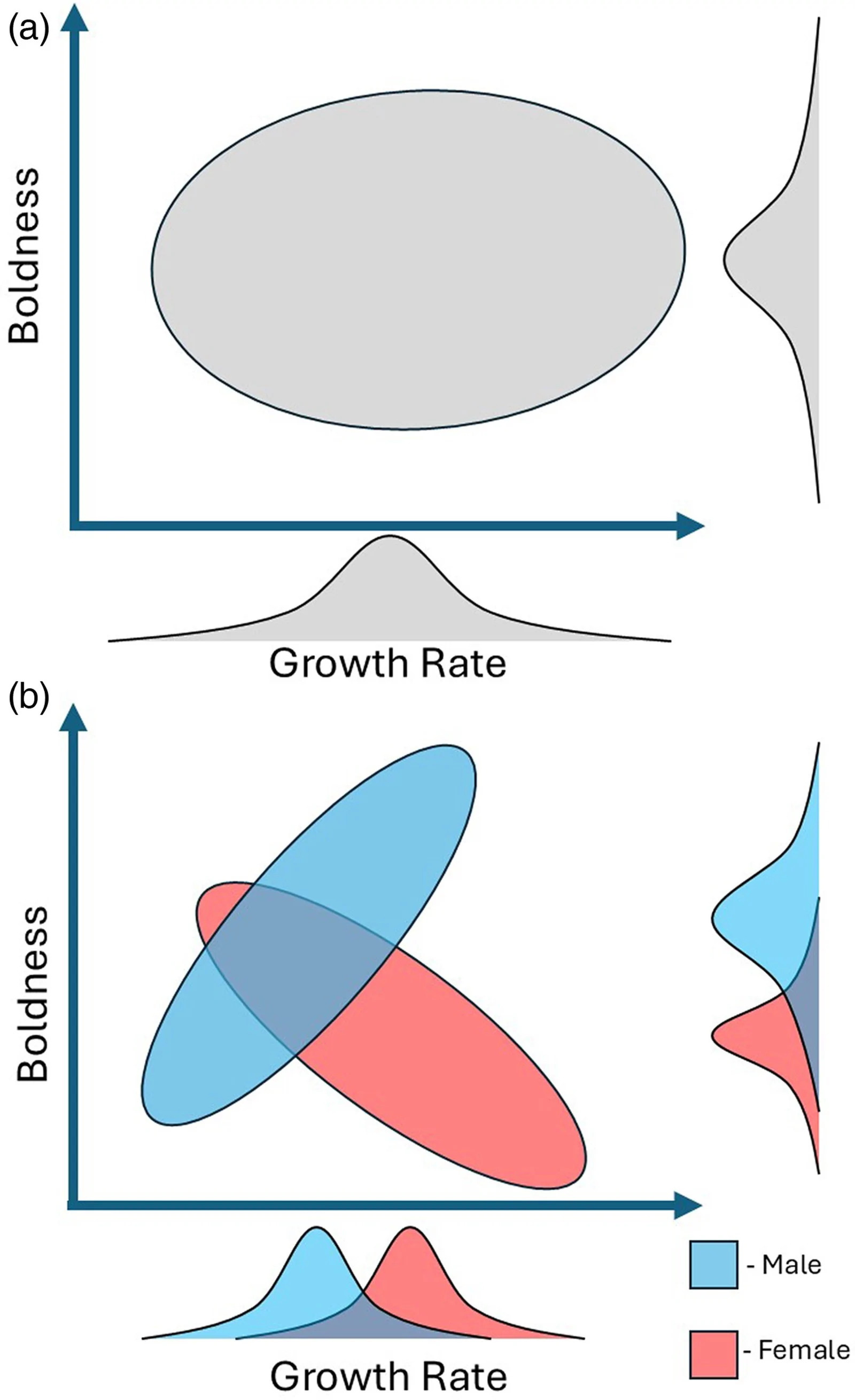
Hypothetical schematic of the POLS hypothesis associations illustrating how consideration of sex-specific correlations might explain the generally weak and null correlations in the literature that ignore sex differences. Panel (a) shows a weak or nil among-individual correlation between boldness and growth rate when ignoring sex, whereas in (b), there may be “hidden” divergent among-individual relationships between the females and males. These plots are for illustrative purposes and not based on real data.

**Figure 2:**
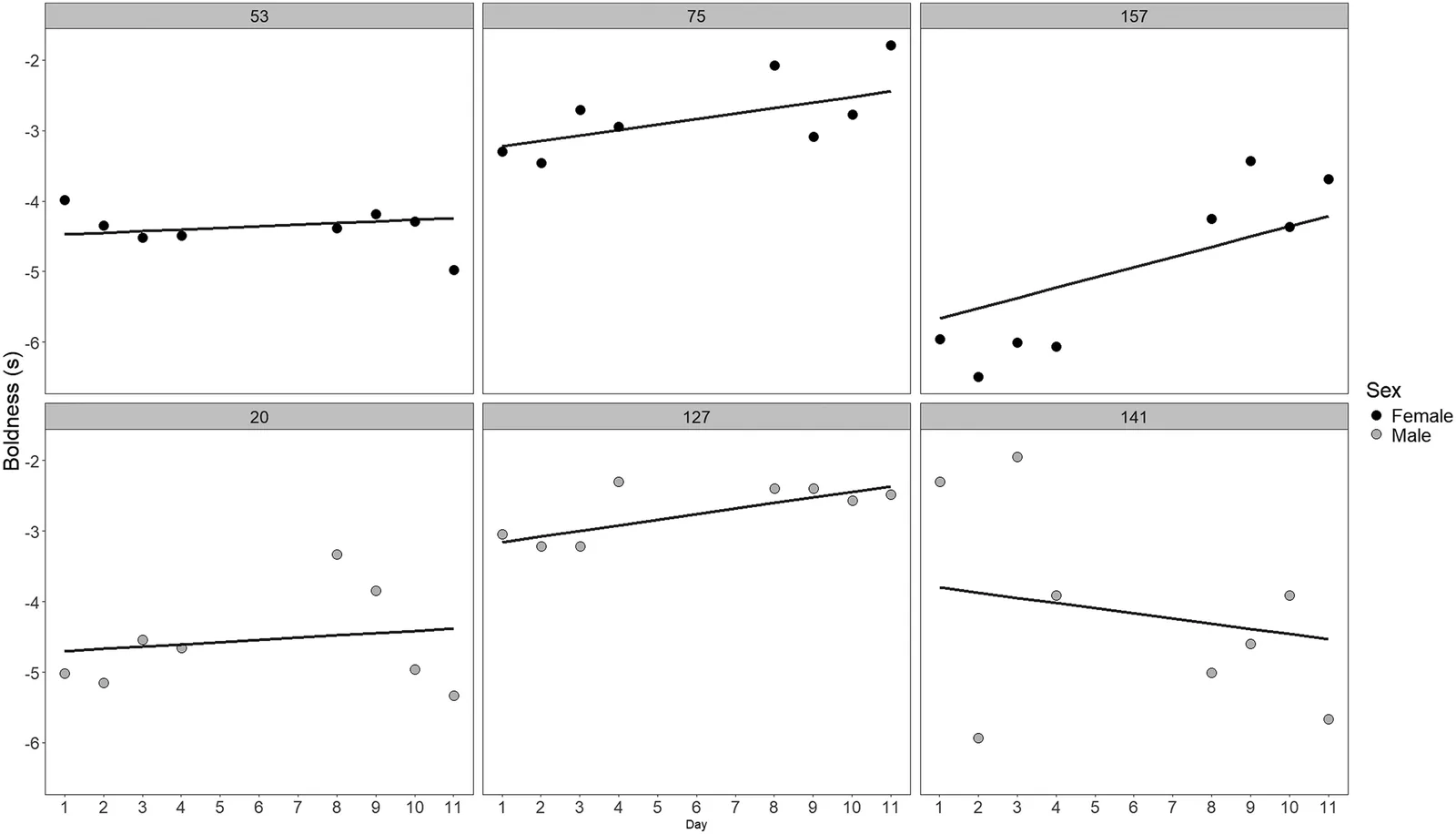
Plots of individual pillbug boldness after log transformation of inverse latency (log-(1/Latency (s)) scores over time. Shown are 6 individuals that highlight a range of responses observed across 11 d of assays. Plot numbers designate individual animal identification numbers. Female trends are presented in the upper three panels, male trends are presented in the lower three panels.

Darwin (1871) initially proposed that sexual selection is responsible for maintaining greater trait variation in males than females. This has been supported regarding morphological traits (Reinhold and Engqvist 2013; Wyman and Rowe 2014; Zajitschek et al. 2020), while evidence for greater variability in male behavior is weaker (Tarka et al. 2018; Harrison et al. 2022; Del Giudice and Gangestad 2023). Currently, a scant few studies show that correlations between physiological and behavioral traits can differ between females and males (Lantová et al. 2011; Krams et al. 2014; Šíchová et al. 2014; Videlier et al. 2019). Perhaps the reason for few studies investigating sex differences in (co-)variances may be due to onerous logistical challenges and complex analyses. Indeed, studying sex-specific trait associations requires large samples of both sexes, substantial repeated sampling effort to achieve precise estimates of individual-level means, and complex analyses (eg see Videlier et al. 2019).

In this study, we investigated sex-specific correlations between productivity (as estimated from growth rate) and boldness behavior using the terrestrial crustacean *Armadillidium vulgare* (hereafter “pillbugs”) as a model species. This species exhibits an unambiguous antipredator behavior (conglobation), is easily individually housed in large numbers, and is amenable to high-throughput behavioral assays (eg Beveridge et al. 2022; Cornwell et al. 2023). Pillbugs have a polygynandrous mating system wherein males can mate with multiple females in a short period, while females can produce multiple broods from a single mating event (Valette et al. 2017; Durand et al. 2020), potentially leading to greater variability in male fitness, which may be maintained via state-dependent differences in risk-taking personality (Dall et al. 2004; Stamps 2007; Biro and Stamps 2008). Pillbugs are indeterminate growers, and females are 20% larger than males (Waller and Verdi 2016; Lefebvre et al. 2019; Beveridge et al. 2022) owing to a faster female growth rate (this study). Specifically, we predict that males will show greater mean boldness, greater among-individual variation in boldness, and lower mean growth rates than females; if one assumes similar growth rate variance between the sexes, then we predict that males will show a positive correlation, and females will show a negative correlation between behavior and growth rate as outlined above (see also Fig. 1b). We assayed boldness in 92 female and 68 male pillbugs 8 times, and mass was measured 4 times over 90 days to assess growth rate. This permitted a sex-specific (co-)variance analysis of behavior and growth using a bivariate mixed-effects model, allowing for sex-specific among- and within-individual variance to be evaluated within a single model. Though complex, we regard this as best practice and provide an annotated R script to encourage others to take up this quantitative approach (see S1).

## Method

### Study species

The common pillbug *A. vulgare* (Crustacea, Isopoda, Oniscidea) is a terrestrial isopod. Native to Europe, *A. vulgare* has a cosmopolitan distribution and is commonly found in south-east Australia (where this study was conducted) (Franklin et al. 2015; Sfenthourakis and Hornung 2018). Pillbugs are found in damp soils as they are highly vulnerable to desiccation and primarily subsist on detritus and fresh vegetation.

Pillbugs show an unambiguous response to predation threat: “conglobation,” whereby the animal rolls its body into a sphere (Horváth et al. 2019). Latency to emerge from conglobation provides an excellent measure of “personality” in *A. vulgare Sensu* Reale et al. (2007), as there is temporally repeatable among-individual variation (Fig. 2; Beveridge et al. 2022; Cornwell et al. 2023; Joynson et al. 2025), and preliminary evidence suggests that boldness values are heritable (A. Joynson, unpublished data).

### Sampling

We collected 200 individual pillbugs between 16 and 21 June 2023 from 5 independent, suburban/urban locations in the Geelong region (Victoria, Australia): (−38.11586319, 144.34268473; −38.10304466, 144.33911704; −38.09409520, 144.32723349; −38.16791324, 144.31825278; −38.19782938, 144.29710151) separated from one another by 1 to 15 km apart. To reduce the likelihood of sampling highly related individuals at each location, animals were collected from 2 to 3 sites separated by a minimum of 20 m and with a maximum of 19 individuals collected per site. In the laboratory, 160 individuals were chosen for the experiment by selecting the smaller individuals from the original sample that would still have considerable scope for growth (female mass range: 0.011 to 0.17 g; male range: 0.022 to 0.17). Following selection and allocation to individual home aquaria, we assessed each for sex: 92 females and 68 males. The 40 animals that were not used for the experiment were released at the site of capture.

### Housing

Animals were housed individually in plastic aquaria (H: 9 cm by L: 25 cm by W: 6 cm). The aquaria floor had a thin layer (1 to 2 cm) of Plaster of Paris, coarsened by sprinkling a small amount of sand on the surface. Sand was added as pillbugs can become overturned and unable to right themselves on the plastic floor, potentially leading to mortality (A.J. pers. obs.). Each aquarium contained 30 g of dry compost (Bannockburn Garden Supplies), which was moistened with deionized water. The compost was pressed to one side of the aquaria to avoid rapid drying and to leave a portion of the Plaster of Paris base uncovered (approx. 15 × 6 cm) to place individuals on and easily observe conglobation latency. The pillbugs readily burrowed into the moistened compost. To assist with moisture retention, white corflute sheeting was placed on top of all aquaria and only removed during behavioral assays, mass measures, to provide food, and when remoistening the compost. Soil moisture was checked visually daily during behavioral assays and weekly during the post-assay period prior to the end of the experiment, and water was added as needed. Each pillbug was provided with mixed frozen vegetables (peas, corn, and carrot) ad libitum. Visual inspection showed consumption of food; excess food was available throughout the experiment, and old food was removed and replaced when it became moldy. The home aquaria for each individual animal were distributed across a temperature-controlled laboratory and were allocated a specific table number to account for spatial/temperature effects present in the laboratory (see below).

### Spatial/temperature effects

The aquaria containing each individual were placed on 1 of 4 tables according to identification number (Table 1: IDs 1 to 40; Table 2: IDs 41 to 80; Table 3: IDs 81 to 120; Table 4: IDs 121 to 160). Individuals selected for each table were done haphazardly and contained a mix of males and females from at least 3 sites. As temperature changes can affect poikilotherm behavior, a proxy of soil temperature was recorded using a water bath placed at the end of each table. Measuring soil temperature directly would have required physically probing the soil, potentially disturbing individuals and confounding behavioral observations. This allowed any temperature differences between tables or between assays to be statistically accounted for when modeling individual behavior. Our laboratory temperatures remained stable throughout the experiment (mean 18.9 °C, range: 17.6 to 20.0 °C).

### Experimental outline

A repeated measures design was used to test for the presence of among-individual (co-)variation in boldness behavior and growth rate. The study took place over a 91-day period. Pillbugs were weighed when first brought into the laboratory between 16 and 21 June, then allocated to individual aquaria with a unique ID number. Animals were then left undisturbed with ad libitum food for a minimum of 35 d (max 40 d) to acclimate to conditions prior to behavioral assays beginning 25 July 2023. Pillbugs were weighed for a second and third time following each round of behavioral assays (25 to 28 July and 1 to 4 August), then weighed a fourth time 1 month later (14 September).

Individual boldness tests were undertaken once daily for 4 consecutive days, which was repeated the following week. Animals were not tested for boldness for 24 h if they were molting, as behavioral assays can harm individuals in this compromised state (A.J. pers. obs.).

To measure boldness, one researcher (always A.J.) simulated a predatory attack on each pillbug. This consisted of gently digging through the compost with tweezers until the individual was located, then gently pushing the individual toward the uncovered portion of the aquaria. Next, the pillbugs were gently pushed 5 times, eliciting conglobation. Conglobated individuals were then raised to a height of approximately 5 cm and dropped onto the uncovered portion of plaster. Latency to unroll (ie boldness) was measured as the time (s) from the individual was dropped until they were completely unrolled and upright or completely unrolled with underside exposed and legs in the air.

### Ethical note

All experimentation was in agreement with the ASAB/ABS Guidelines for the treatment of animals in behavioral research and education, the Australian Code for the care and use of animals for scientific purposes, and the Prevention of Cruelty to Animals Act 1986 (Vic). As this study was conducted in Victoria, Australia, on an invertebrate, no ethics approval was required.

### Statistical analysis

We analyzed behavior and mass data using a bivariate mixed-effects model. Such a model combines 2 linear models, 1 for each response, through a covariance matrix of the random effects on each response. It permits evaluation of covariation between traits at the individual level, for each sex, within a single parsimonious model. Because we were specifically interested in sex-specific responses, intercepts and slopes were fitted separately for each sex at the mean level and at the individual level using random intercepts and slopes. This permitted temporal trendlines of mass and behavior to differ between sexes, and variance around those trends to also differ by sex. Table position and temperature were also added as covariates for mass and boldness, respectively. The following indicates the 2 linear model equations used in our bivariate model analysis, both of which contain random intercepts and slopes for day that were made sex-specific, including sex-specific residual variation—we use notation that is common and familiar to users of R (R Core Team 2025):


(1) $$\mathrm{Mass}=0+\mathrm{tableID}+\log(\mathrm{Day}{)}^{*}\mathrm{Sex}+(1+\log(\mathrm{Day})|\mathrm{ID},(\mathrm{group},\;\mathrm{by}=\mathrm{Sex}))+\mathrm{sigma}=0+\mathrm{Sex}.$$

(2) $$\mathrm{Boldness}=0+\mathrm{temp}+{\mathrm{Day}}^{*}\mathrm{Sex}+(1+\mathrm{Day}|\mathrm{ID},\;(\mathrm{group},\;\mathrm{by}=\mathrm{Sex}))+\mathrm{sigma}=0+\mathrm{Sex}.$$

For each linear model, there is an associated linear model specifying the residual variation, sigma. In all models and submodels, we use the term “0+” to remove an overall intercept (reference group) and thus fit group intercepts directly rather than a reference group and deviations from it to specify other groups. This is done for ease of interpretation and comparison of groups. We report our mean-level trends and random-effects-level correlations between boldness and growth for males and females in detail, as these were the primary interest of this study. Full model output, including correlations we do not explore (ie nitial mass-boldness), is included in S2.

We used the R package brms (Bürkner 2017) as a highly flexible package that facilitates building structured covariance matrices by sex (by using the “group” function) and sex-specific residual variances (where sigma, the residual, is a linear model with an intercept and sex as a predictor variable). The model was fitted using default priors. Model estimates are provided with their 95% credible intervals (CrI). The model was run on 4 chains of 7,500 iterations with a warm-up of 500 iterations. Diagnostic checks were performed to ensure appropriate model convergence ($\hat{R}$ = 1 ± 0.001, bulk and tail effective sample sizes all >1,000). Random intercepts and slope predictions (the BLUPS) followed a normal distribution (Zuur et al. 2009). We used the posterior predicted values from the model to estimate differences between means and variances between females and males by calculating a difference value at each iteration to yield a distribution from which we obtained the mean difference and 95% credible intervals. We provide annotated code with a guide on how to perform data checks and analyses in the supplemental material (S1).

When assessing the fit of exploratory univariate models of boldness, we found there was a high level of collinearity between table position and temperature (as tables were not moved and table position also captures temperature variation). As temperature is known to strongly affect ectotherm behavior (Joynson et al. 2025), we decided to remove table position from the model and retain temperature. For the mass model, we retained only table position, as that variable better captures long-term temperature variation that might affect growth rates.

Boldness was measured as the inverse of latency to unroll (1/Latency) in seconds. Raw boldness was highly skewed; a log transformation of boldness removed the dependency between mean and variance, ensured linearity of individual trends across days, and ensured that residuals were approximately normally distributed. Raw mass values were also skewed; a square-root transformation of mass and a log transformation of day linearized the relationship and ensured that residuals were approximately normally distributed.

When modeling boldness, the predictor “day” was mean-centered, so intercepts are estimated at the midpoint of the experiment, a useful representation for individual predictions for individual means. When modeling mass, the predictor of log-day was left-centered, so intercepts represent mass values at the beginning of the experiment (rather than at Julian day 0, which would affect the estimation of the intercept–slope covariance); left-centering was used to meaningfully account for the fact that scope for future growth decreases with mass, given that we could not closely control for starting mass, and this is accounted for by the intercept–slope covariance. Given mean centering of boldness, among-individual correlations from our bivariate model represent correlations between growth and behavior at the midpoint of the experiment.

Finally, we estimated trait repeatability for boldness and mass to provide an indication of how distinct and predictable individual differences were. Given that there was evidence that individuals had differing temporal trendlines with varying slope (individual variation in habituation and individual variation in growth), this means that repeatability is not constant over time because individual variance is changing through time; hence, we estimated R evaluated at different time points using standard equations and using the posterior predicted values to obtain CIs (Biro and Stamps 2015; Stamps and Biro 2023); see also S1 for coding).

## Results: mean-level trends

Females and males showed similar increases in boldness over the course of the experiment, with very similar estimates of female and male habituation and overlapping confidence intervals (β_Female-day_ = 0.06, CrI: 0.04, 0.08, β_Male-Day_: 0.06, CrI: 0.03, 0.09; Fig. 3). Males appear slightly more bold than females; however, overlapping credible intervals indicate there were no significant differences in boldness at the midpoint of behavioral assays (β_Male-intercept_: −4.87, CrI: −6.62, −3.15, β_Female-intercept_: −5.06, CrI: −6.79, −3.35). Boldness increased with temperature, as expected in poikilotherms; however, this effect did not reach significance (β_Temp_: 0.06, CrI: −0.02, 0.16).

**Figure 3:**
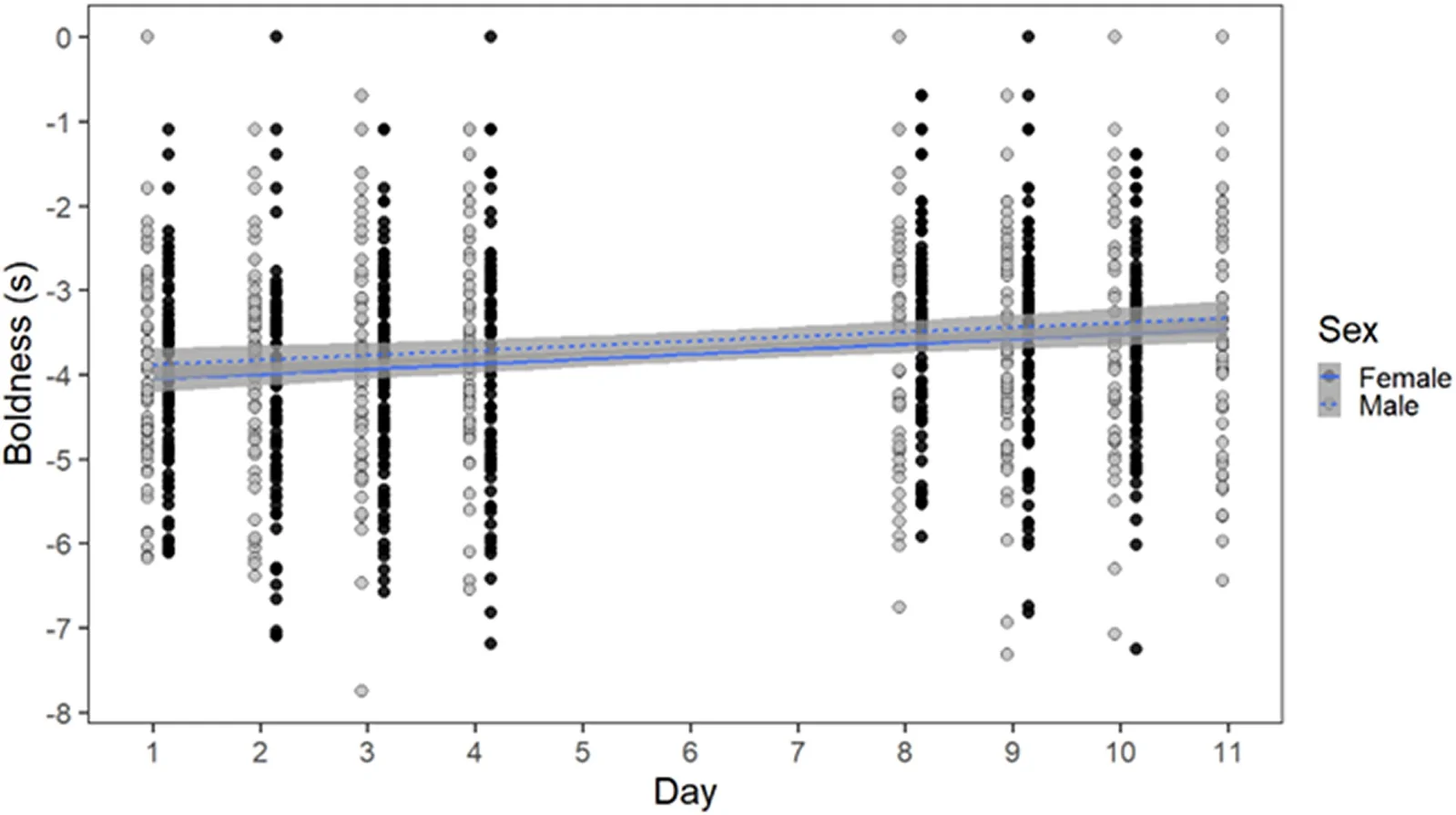
Mean-level trends of boldness in male (light circles, dashed line) and female (dark circles, solid line) pillbugs over the course of 11 d of behavioral tests. Boldness (inverse latency) has been log-transformed; see methods for details.

Female and male boldness responses were both substantially repeatable at the midpoint of the experiment (female *R*_day6_ = 0.65, CrI: 0.57, 0.73; male *R*_day6_ = 0.65, CrI: 0.55, 0.74), indicating that individual differences were relatively distinct. The sexes were similarly predictable in their behavior, as residual variation estimates were very similar (female boldness residual: −0.30, CrI: −0.36, −0.23; male boldness residual: −0.25, CrI: −0.32, −0.17). We detected a positive intercept–slope correlation in boldness behavior among males, indicating that individuals were becoming increasingly different through time (*r* = 0.40, CrI: 0.06, 0.70; Fig. 4). As males showed increasing among-individual variation over time, repeatability increased to a maximum at day 11 (*R*_day11_ = 0.72, CrI: 0.63, 0.80). By contrast, female trendlines were largely consistent through time, as given by the trivial intercept–slope correlation among females (*r* = 0.06, CrI: −0.31, 0.45), and hence repeatability did not change with time (*R*_day 11_ = 0.67, CrI: 0.58, 0.75).

**Figure 4:**
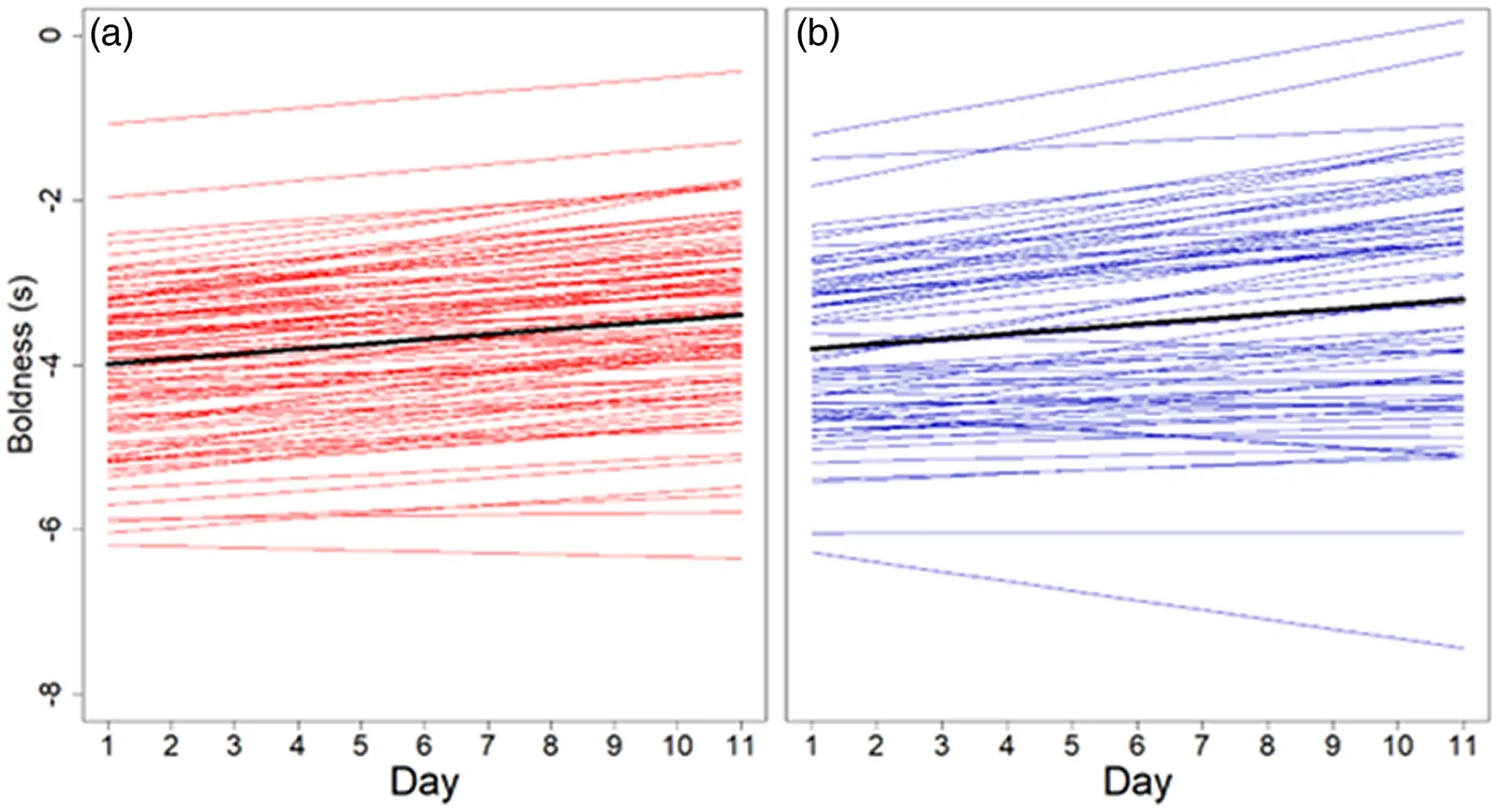
Behavioral reaction norms for pillbug females (a, left) and males (b, right) over the 11 d of boldness tests. Thick black bars are mean trends. Boldness values are log-transformed inverse latency (s).

Although there were no significant differences in mass between females and males at the beginning of the experiment (β_Female_ = 0.21, CrI: 0.18, 0.23; β_Male_ = 0.24, CrI: 0.21, 0.26, Fig. 4), females had higher growth rates (β_Female-Growth_: 0.028, CrI: 0.025, 0.032; β_Male-Growth_: 0.019, CrI: 0.016, 0.022). There was some significant variation in mass with regard to room position, and this was included to account for any systematic mass differences based on sample allocation within room (β_Table4_ = 0.05, CrI: 0.02, 0.08; all others NS).

As expected for a non-labile trait, mass values were highly repeatable among males and females when evaluated at the midpoint of the experiment (female mass *R* = 0.98, CrI: 0.97, 0.99; male mass *R* = 0.95, CrI: 0.92, 0.97, Fig. 5). As indicated by high *R* values, there was a very small amount of residual variation in mass, and this was very similar between females and males (σ-_Female_: −4.427, CrI: −4.53, 4.31, σ-_Male_: −4.31, CrI: −4.44, −4.17). As repeatability of boldness and mass were both quite high, and differences between individuals in both behavior and growth were largely maintained throughout the duration of the experiment, this suggests that boldness and growth rate might be correlated at an individual level, which we explore in detail next.

**Figure 5:**
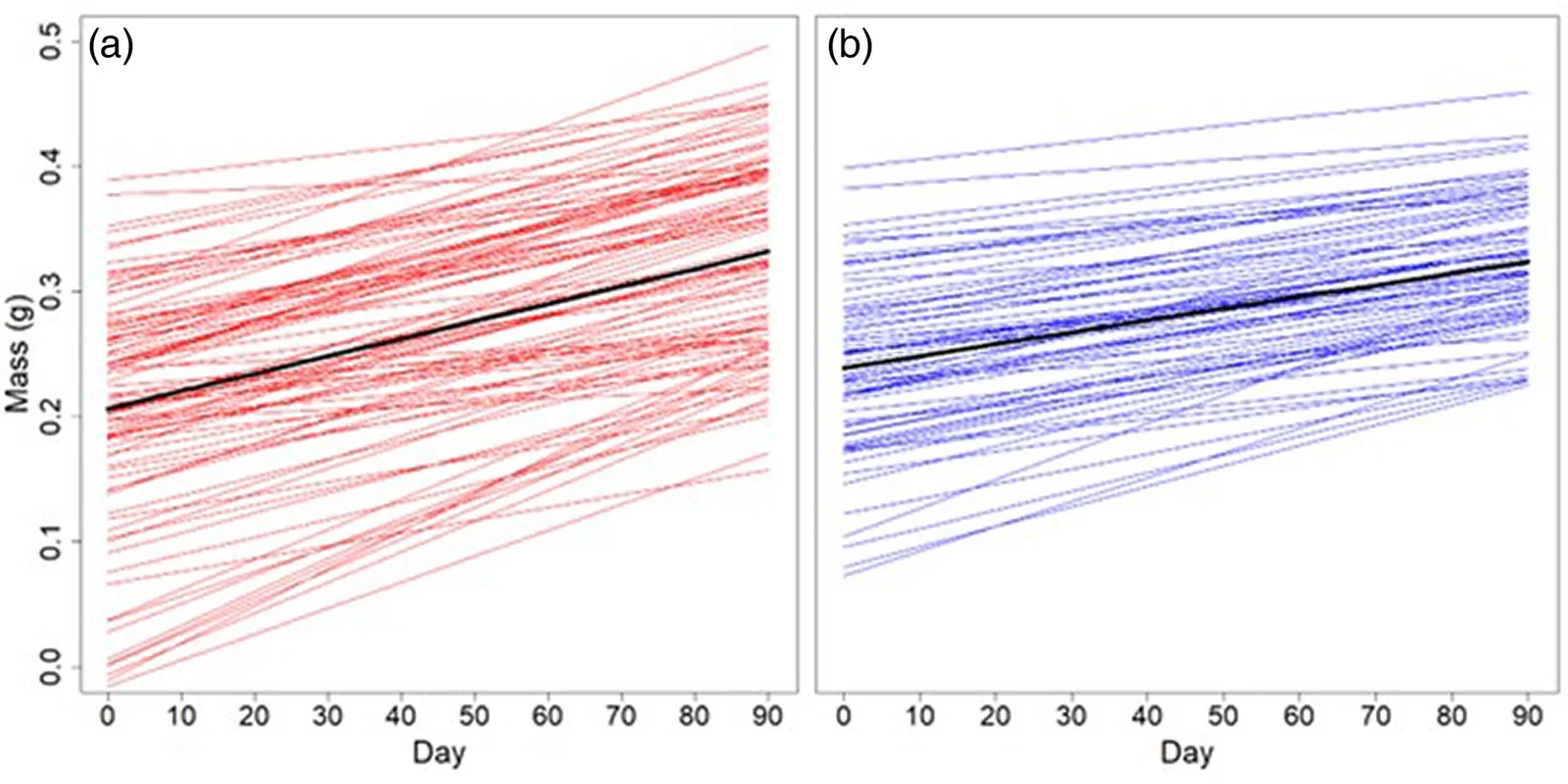
Growth trajectories for individual pillbug females (a, left) and males (b, right). Mean growth trends are depicted by a thick black trendline. Mass (g) values have been square root transformed.

**Figure 6:**
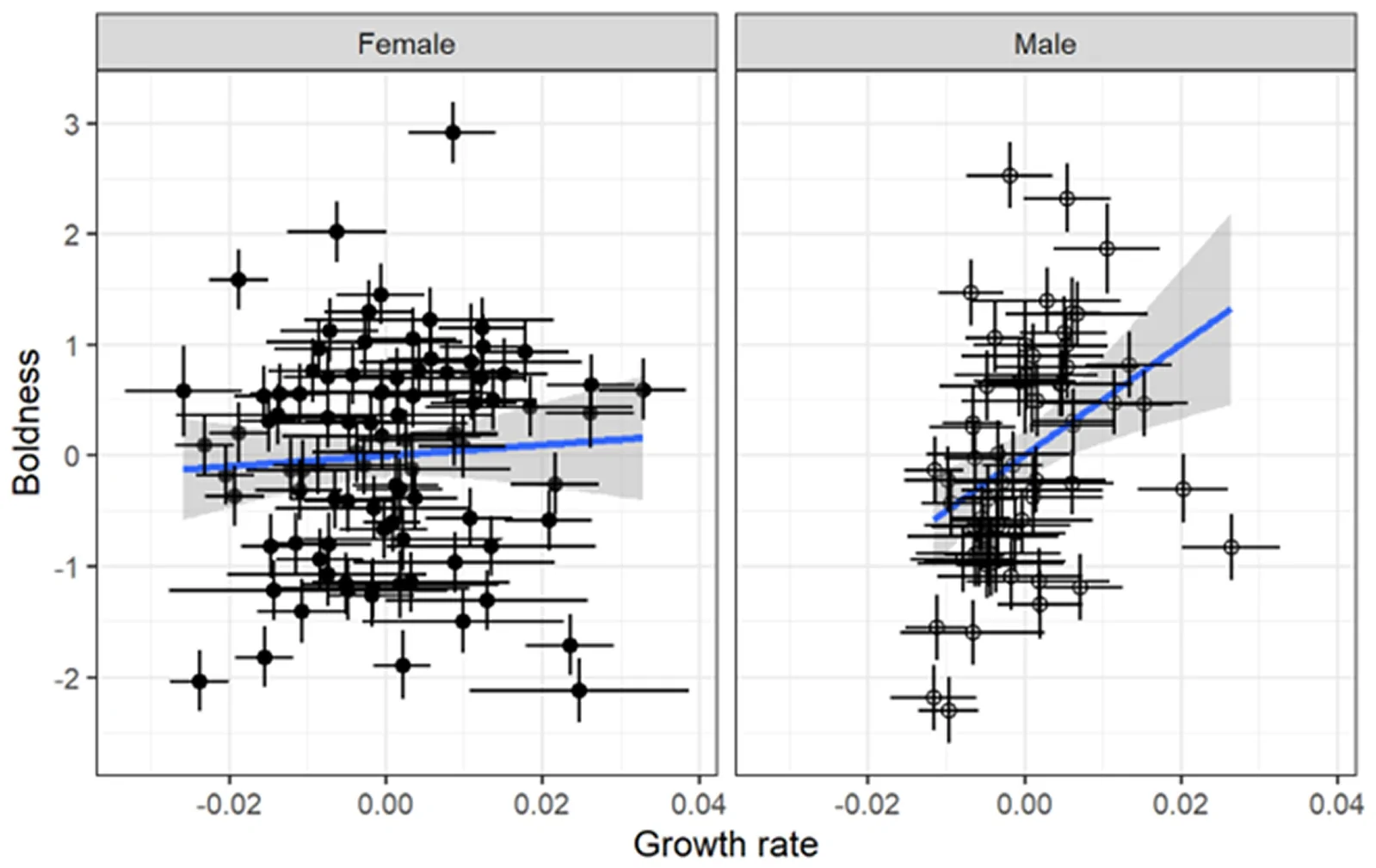
Covariance of boldness(s) and growth rate among pillbug females and males. Estimates and SE values are from model predictions of the random effects.

## Individual-level trends

### Boldness

Boldness responses at the midpoint of the experiment showed a substantial variation in both females (Female-σ_Day 6 intercept_: 1.01, CrI: 0.86, 1.19, Fig. 4a) and males (Male-σ_Day 6 intercept_: 1.07, CrI: 0.89, 1.29, Fig. 4b), with no difference in variation between sexes (Mean diff_Day 6_: −0.13, CrI: −0.73, 0.40). How individual boldness changed over the duration of the experiment initially appeared to be sex-specific, with females showing slightly lower variation in slopes (Females-σ_slope_: 0.05, CrI: 0.01, 0.08) than males (Male-σ_slope_: 0.08, CrI: 0.05, 0.12), but post hoc analysis showed that this was not statistically significant (Mean diff: <−0.01, CrI: −0.01, 0.002). Males showed a positive intercept–slope correlation (male *r*_int-slope_: 0.40, CrI: 0.06, 0.71, Fig. 4b), whereas females showed no intercept–slope correlation (female *r*_int-slope_: 0.06, CrI: −0.31, 0.45). When evaluated using the mean-centered estimates of boldness, there was uncertain evidence for greater intercept–slope covariance among males than females (Mean diff: −0.03, CrI: −0.07, 0.005).

### Growth

There was a small but significant amount of variation in growth rate (ie individual variation in slopes) among females (SD = 0.015, CrI: 0.012, 0.018) and among males (SD = 0.010, CrI: 0.007, 0.013). Females did show slightly greater variance in growth, but this was extremely small (Mean diff: <0.001, CrI: <0.0001, <0.01). Both sexes showed negative correlations between initial mass and growth, while this correlation appeared stronger in males (female *r* = −0.58, CrI: −0.73, −0.40; male *r* = −0.67, CrI: −0.83, −0.44); a post hoc comparison showed there was no significant difference in the allometry of males and females (Mean diff: 0.08, CrI: −0.19, 0.31).

As there was substantial among-individual variation in both boldness and growth rate in both sexes, we next report the covariation between individual boldness and growth in a sex-specific manner, allowing an assessment of sex-specific pace-of-life relationships.

### Correlation between boldness and growth

Among females, there was no correlation between growth rate and boldness (female *r*_Intercept-Slope-Day6_: 0.05, CrI: −0.20, 0.30, Fig. 6). Among males, there was evidence of a weak correlation between growth rate and boldness (male *r*_IS-Day6_: 0.25, CrI: −0.06, 0.53, Fig. 6). However, a post hoc comparison showed there was no difference between male and female correlations (Mean diff. = −0.19, CrI: −0.58, 0.20). If one evaluates these correlations using predicted intercepts at the end of the study (see S1; also Stamps and Biro 2023), when males showed greater variance, results are similar (female *r_IS-Day11_*: 0.03, CrI: −0.24, 0.29; male *r_IS-Day11_*: 0.25, CrI: −0.06, 0.53), but they were also not significantly different (Mean diff. = −0.22, CrI-0.61, 0.19).

## Discussion

The intention behind this study was to refine predictions from the extended POLS hypothesis (Stamps 2007; Biro and Stamps 2008; Careau et al. 2008; Biro et al. 2010; Réale et al. 2010) by incorporating sex as a significant variable, which is likely to influence correlations between physiological traits and animal personality (Hämäläinen et al. 2018; Immonen et al. 2018), and using and highlighting bivariate mixed models with fixed and random effects made sex-specific to evaluate sex differences in those correlations as best practice analysis methods (see Supplement). We found there was some evidence for faster-growing males to be bolder, as predicted, but there was no correlation in females—there were no differences in boldness, but females grew faster and were larger as expected.

We begin by discussing our findings at the mean level. Because male pillbugs show greater dispersal distances than females as a means to locate unrelated females (Verne et al. 2012; Fortin et al. 2018; Durand et al. 2019; Durand et al. 2015), we predicted that males would show greater mean boldness than females; however, we found no difference in mean boldness between males and females. That males and females showed similar mean-level boldness is similar to findings of Zamora-Camacho (2023), who found similar boldness in male and female *A. vulgare* in response to different threat conditions. We also observed that males and females habituated to the assays and that they did so at similar rates. Similar rates of habituation among sexes to a stressor are reminiscent of Houslay et al. (2019), who found that *Poecilia reticulata* showed similar habituation rates of hormonal response between males and females, but this was not related to any feature of their reproduction or ecology. Reviews of habituation patterns emphasize the complex nature of habituation patterns generally (Rankin et al. 2009), and how these patterns differ by sex is likely to be trait-specific and will continue to be a fruitful area of study (Eberhard 2024). Finally, residual variation (inverse predictability) did not differ between males and females for boldness nor for mass; we did not have any predictions on this, and we have not found anything in the literature discussing how or why predictability in behavior might differ between sexes. As for differences in the life-history trait of growth rate, we found that females grew faster than males—this is consistent with previous studies, including our own, when pillbugs are held in the laboratory (Waller and Verdi 2016; Lefebvre et al. 2019; Beveridge et al. 2022).

Next, we discuss the extent of among-individual variation and whether it differs among sexes. We found that among-individual variation in both intercepts and slopes (habituation) was similar between males and females. There was a positive intercept–slope correlation among males, increasing variance between individual males over time (Fig. 4b). As variation in male boldness clearly increased, we evaluated repeatability for both sexes at the midpoint of the study (both *R*_day 6_ = 0.65) and at the end of the experiment when among-individual variation in male boldness was highest but unchanged in females (male *R*_day 11_ = 0.72; female *R*_day 11_ = 0.65). Despite this, individual variation among males was still not significantly greater than variation among females at day 11, similar to other studies showing no sex differences (Westneat et al. 2015; Mitchell et al. 2021). Thus, our study provides little support for the greater male variability hypothesis (Harrison et al. 2022). However, Darwin's (1871) original formulation of the greater male variability hypothesis referred specifically to sexually selected morphological traits. Future studies of the greater male variability hypothesis regarding behavioral traits require careful consideration of prior selection pressures shaping male and female behavior (Andersson 1994; Shuster and Wade 2019; Zajitschek et al. 2020).

Finally, and most importantly, we discuss sex-specific associations between life history and behavior. As males provide no post-copulatory offspring investment and mating competition favors larger males, we predicted that males would show a positive correlation between individual growth rate and boldness. This was supported, though the correlation was not large and was uncertain. We predicted that females are likely to show asset protection, being that they are capital breeders and reproductive success is greater for faster-growing (and hence large) individuals, evident as a negative correlation with boldness. We saw no evidence of this, with zero correlation appearing to be the case. Both male and female pillbugs showed an expected pattern of converging mass as individuals approached their asymptotic mass. This negative correlation was stronger in males, nearly approaching significance. Males approaching their asymptotic mass earlier than females aligns with sex-specific predictions of allocation trade-offs. Males have morphological and behavioral features suggestive of scramble mating tactics, including larger male antennae for chemoreception (Lefebvre et al. 2000) and greater male dispersal (Durand et al. 2019). As larger females produce more offspring (Waller and Verdi 2016; Antoł and Czarnoleski 2018) and females can store sperm for over 1 year, this should encourage an extended growth period in females following maturation (Suzuki and Ziegler 2005). This combination of scramble mating tactics in males and mass–fecundity relationship among females suggests male fitness is dictated predominantly by access to females, whereas female fitness is more strongly influenced by female mass. Together, these selective pressures would promote maturation at smaller sizes in males and a stronger convergence to an optimal mass asymptote than among females.

Both our study and Harrison et al. (2022) demonstrate that tests of the greater male variability hypothesis must consider the species-specific relationship between trait variability and sexual selection when making predictions. Further, a meta-analysis of sex differences in variability conducted on mice shows that even within a species, variability may be higher in males or females depending on the trait being studied (Zajitschek et al. 2020), emphasizing the need for greater understanding of species- and trait-specific evolution when making predictions of variability between sexes. This becomes most apparent regarding sex-specific residual variability or “predictability” (Cleasby et al. 2015;  Westneat et al. 2015; Mitchell et al. 2016). Some empirical work has described sex-specific differences in predictability (Brand et al. 2023a; Kelly 2024; Stingo-Hirmas et al. 2025), and our study found no sex difference in predictability, but at present, the literature on sex-specific differences in predictability is largely descriptive.

Research into the sex-specific nature of POLS correlations may benefit from investigating boldness and dispersal behaviors in pillbugs and assessing these sex-specific (co-)variances in a multivariate framework to assess whether a behavioral syndrome (Sih et al. 2004) exists between boldness and exploration in males to increase the likelihood of finding and securing mates. Currently, there is little evidence available to indicate whether females would also present a behavioral syndrome. This raises the possibility of divergence between females and males in trait correlations, as discussed by Hämäläinen et al. (2018). This is most likely to occur in species with very different reproductive roles, but it requires that fitness-optimizing trait combinations differ between males and females and that the genetic architecture of relevant traits permits the sexes to independently respond to selection (Immonen et al. 2018). For instance, males that provide limited parental care (as in pillbugs) may achieve higher fitness with a combination of rapid maturation, boldness, and exploration behaviors, whereas females may achieve higher fitness with a combination of somatic growth, sociability, and investment in immune defenses.

In conclusion, our study adds to a nascent body of work exploring how among-individual correlations between life history and personality can differ between the sexes (Krams et al. 2014; Videlier et al. 2019; Yarwood et al. 2021; Brand et al. 2023b). To advance our understanding of animal personality, future studies ought to employ designs that address the statistical requirements for sex-specific analyses. These designs will be challenging, especially given the data requirements for more complex statistical models. However, the foundational role of sex in shaping life history and behavior among sexually reproducing species means this research avenue will almost certainly advance our understanding of animal personality and its integration with physiology and life history.

## Supplementary Material

arag116_Supplementary_Data

## Data Availability

Analyses reported in this article can be reproduced by using the data and code provided by Joynson et al. (2026).
