# Supplementary material for: Does (co-)variation between boldness and growth differ between sexes? A nuanced and powerful evaluation of the pace of life hypothesis
Source: Behav Ecol. 2026 Oct 21;37(6):arag116. doi: 10.1093/beheco/arag116 (PMC13684408; doi:10.1093/beheco/arag116)
Supplement: Supplementary file 1 [file arag116_supplementary_data.zip › S2 Full Bivariate Model Output.docx]

Joynson et al. 2026 – Does (co-)variation between boldness and growth differ between sexes?

Supplement 2: Complete model output from hierarchical bivariate model of Boldness and Mass of male and female Armadillidium vulgare.
